# Detection of Potential Zoonotic *Bartonella* Species in African Giant Rats (*Cricetomys gambianus*) and Fleas from an Urban Area in Senegal

**DOI:** 10.3390/microorganisms10030489

**Published:** 2022-02-22

**Authors:** Jean-Paul Demoncheaux, Hacene Medkour, Meriem Louni, Laurie Laugier, Christelle Pasqualini, Florence Fenollar, Bernard Davoust, Oleg Mediannikov

**Affiliations:** 1 Animal Epidemiology Expert Group of the Military Health Service, 37100 Tours, France; jp.demoncheaux@gmail.com (J.-P.D.); bernard.davoust@gmail.com (B.D.); 2 Military Health Service, French Armed Forces in Senegal, Dakar 18524, Senegal; christelle.pasqualini@gendarmerie.interieur.gouv.fr; 3 IRD, AP-HM, MEPHI, Aix Marseille University, 13005 Marseille, France; hacenevet1990@yahoo.fr (H.M.); louni_meriem@yahoo.fr (M.L.); 4 IHU Méditerranée Infection, 13005 Marseille, France; laugier.laurie@yahoo.fr (L.L.); florence.fenollar@univ-amu.fr (F.F.); 5 IRD, AP-HM, SSA, VITROME, Aix Marseille University, 13005 Marseille, France

**Keywords:** African giant rat, *Bartonella*, *Cricetomys gambianus*, flea, Senegal, *Xenopsylla cheopis*, zoonosis

## Abstract

Bartonellae are bacteria associated with mammals and their ectoparasites. Rodents often host different species of *Bartonella*. The aim of this study was to investigate the presence of *Bartonella* spp. in African giant pouched rats (*Cricetomys gambianus*) and their ectoparasites in Dakar, Senegal. In 2012, 20 rats were caught, and their fleas were identified. DNA was extracted from 170 selected fleas and qPCR was carried out to detect *Bartonella* spp. Subsequently, a *Bartonella* culture was performed from the rat blood samples and the isolated strains (*16S rRNA*, *rpoB*, *ftsZ* and ITS3) were genotyped. A total of 1117 fleas were collected from 19 rats and identified as *Xenopsylla cheopis*, the tropical rat flea. *Bartonella* DNA was detected in 148 of 170 selected fleas (87.1%). In addition, *Bartonella* strains were isolated from the blood of 17 rats (85%). According to *Bartonella* gene-sequence-based criteria for species definition, the isolated strains were identified as *B*. *massiliensis* (four strains) and two potential new species related to the zoonotic *B*. *elizabethae*. In this paper, these potentially new species are provisionally called *Candidatus* Bartonella militaris (11 strains) and *Candidatus* Bartonella affinis (two strains) until their description has been completed. *Cricetomys gambianus* and its fleas could constitute a public health risk in Dakar due to the high prevalence of *Bartonella* infection reported.

## 1. Introduction

*Bartonella* is the only genus of bacteria belonging to the family *Bartonellaceae*. These microorganisms are Gram-negative and facultative intracellular bacteria. Currently, more than 40 *Bartonella* species have been described. Many of them can infect a large range of different mammal species, often rodents, which are regarded as the main reservoir hosts of many species of these bacteria. At least 20 species of *Bartonella* are rodent-associated [1] and more than ten species are zoonotic and cause human diseases [2]. The most common pathogens responsible for human bartonellosis are *Bartonella bacilliformis*, *Bartonella quintana* and *Bartonella henselae*; these species are not associated with *Rodentia*. However, many small rodent-associated bartonellae have been reported to cause human diseases. *Rattus*-associated *B*. *elizabethae*, *B*. *washoensis* and *B*. *vinsonii arupensis* may cause culture-negative endocarditis, *B*. *grahamii* is suspected to induce neuroretinitis and endocarditis and *B*. *tribocorum* may cause chronic bacteriaemia [3,4]. Blood-sucking arthropods, such as fleas, ticks, sand flies and lice, can transmit different *Bartonella* species [5].

The African giant rat (*Cricetomys gambianus*), also known as the Gambian pouched rat, is a large rodent in the exclusively African family *Nesomyidae*. This is one of the largest rodents in the world. It weighs between 1.0 and 1.4 kg and grows to approximately 0.9 m long, including the tail, which makes up half of its total length [6]. This animal is found in sub-Saharan African countries, from Senegal to Kenya, and in Southern African countries, from Angola to Mozambique. *Cricetomys gambianus* is widespread in different natural biotopes and can frequently be found in semi-urban places, near human habitations. This species is nocturnal and eats fruit, seeds and small animal-like insects. In many African countries, especially in West Africa, giant pouched rats are considered as an important source of meat and are hunted by local populations [7]. In Senegal, *C*. *gambianus* is a commensal animal and one of the most common rodents encountered in the city of Dakar.

Wild rodents are well known to host abundant numbers of parasites. Several studies have mentioned the large number of ectoparasites encountered in *Cricetomys gambianus*, including several species of fleas [7]. To date, fleas have been considered as the main players in the *Bartonella* genus epidemiology due to the high variety and prevalence of *Bartonella* species and strains detected on fleas, their potential role of complementary reservoirs and their efficiency in transmitting these bacteria between rodent populations [8]. Commensal rodents and their fleas infected with *Bartonella* species can facilitate the transmission of these pathogens to humans due to, on the one hand, the abundance and close association of rodents with humans and, on the other, the frequency with which fleas eat and are able to move. In Senegal, knowledge of the distribution and epidemiology of *Bartonella* in rodents and ectoparasites is limited and the role of rodent-associated bartollenosis in human public health is still poorly understood by health professionals.

Here, we investigated the occurrence of *Bartonella* spp. infections in African giant pouched rats trapped at an urban location in Dakar, Senegal and in their fleas. We also characterised the potential new *Bartonella* species and strains isolated from these rats.

## 2. Materials and Methods

### 2.1. Ethics Statement

This study was carried out as part of the rodent control measures aimed at maintaining a sufficient level of hygiene and preserving public health in French military camps. The study protocol was written in accordance with European regulations on the protection of animals used for scientific purposes. Permission to set traps in the study area was granted by the French Armed Forces Command in Senegal. All animals were treated in a humane manner and in accordance with previous regulations.

### 2.2. Study Site and Sample Collection

In 2011 and 2012, rodent trapping was conducted for many weeks, within a French military camp located in Ouakam (14°42′0″ N; 17°28′0″ E), a residential quarter of Dakar, Senegal. Metal cages were placed outdoors, in areas surrounding homesteads, in deep gutters and rainwater harvesting systems. These traps were checked for the presence of rodents every day, early in the morning. Each cage containing a rat was transported to the French military veterinarian at the camp, who identified the rat and collected the ectoparasites.

A system was used to restrain the animal. After restraining it, each rodent was anesthetised by an intramuscular ketamine injection (Imalgene 1000^®^, Boehringer Ingelheim, Lyon, France). Whole blood was collected by cardiac puncture in an EDTA-coated tube.

Each rat was brushed with a comb to collect ectoparasites onto a large sheet of white paper. Ectoparasites were placed in tube containing 70% alcohol. Each tube was identified according to the host from which parasites were collected. All the samples (blood and ectoparasites) were stored (at −20 °C and room temperature, respectively) and transported two weeks later to the IHU Méditerranée Infection, Marseille, France. In the IHU, ectoparasites were stored at −20 °C and blood samples at −80 °C until analysis.

Within the framework of the Nagoya accord application, we have obtained authorization to access and use, in France, genetic resources from captured rodents (authorization N°001042 from the Ministère de l’environnement et du développement durable, Dakar, Senegal).

### 2.3. Ectoparasite Identification

All ectoparasites were morphologically identified using identification keys. Each one was photographed using a Stereo Discovery V20 stereomicroscope. The ectoparasites’ genus and species were then confirmed for some specimens, selected randomly, by using primers targeting the *18S* rRNA gene [9].

### 2.4. DNA Extraction and Screening for Bartonella *spp.* by the Real-Time PCR (qPCR)

DNA was extracted individually from selected ectoparasites, as well as from all rat blood samples. Extraction was performed using the commercial EZ1^®^DNA tissue kit, (Qiagen, Courtaboeuf, France) and was performed on a BIOROBOT EZ1 (Qiagen, Courtaboeuf, France) per the manufacturer’s instructions. Prior to DNA extraction, ectoparasites or 200 μL of rat blood was digested with proteinase K and incubated at 56 °C overnight.

DNA was eluted in 200 μL of distilled water and stored at −20 °C until analysis. The qPCRs were prepared and performed in 20 μL final volume containing 10 μL of Master Mix Roche (Roche Diagnostics, Meylan, France), 0.5 μL (of 20 μM concentration) of each primer, 0.5 μL of the probe (of 5 μM concentration), 0.5 μL UDG, 3 μL of distilled DNAse- and RNAse-free water and 5 μL of the DNA sample. The assay was performed in a CFX96 Real-Time system (BioRad Laboratories, Foster City, CA, USA) using the protocol: one incubation step at 50 °C for two minutes and an initial denaturation at 95 °C for five minutes, followed by 40 cycles of denaturation at 95 °C for ten seconds and annealing and extension at 60 °C for 30 s. Positive (known *Bartonella* DNA) and negative (only mixture) controls were added in each reaction.

The screening for *Bartonella* spp. was carried out using genus-specific qPCR targeting the internal transcribed spacer 3 (*ITS3*) region for the selected ectoparasites and all rat blood samples. Positive samples were screened again using the *ITS2* qPCR (Table 1). Only positive samples by both systems were considered as positive.

### 2.5. Bartonella *spp.* Culture and Isolation and MALDI-TOF MS Identification

Subsequently, rat blood samples were plated on Columbia agar with 5% sheep blood (COS). Plates were incubated at 37 °C with 5% CO_2_ and checked for the growth of bacterial colonies morphologically compatible with *Bartonella* species every 12 h for seven days. Isolates were verified as *Bartonella* species using PCR and sequencing of the *16S rRNA*, *rpoB* and *ftsZ*, as well as the ITS partial genes (Table 1). In parallel, they were identified by MALDI-TOF mass spectrometry. The obtained spectra were imported into MALDI Biotyper 3.0 software (Bruker, Billerica, MA, USA) and analysed against the main spectra of bacteria included in two databases (Bruker, as well as Microbes Evolution Phylogeny and Infections (MEPHI), which is constantly updated).

### 2.6. Genetic Amplification by Standard PCR, Sequencing and Phylogeny

All of the selected fleas were suggested to the 18S-based standard PCR. *Bartonella* isolates were tested by PCRs for species identification using the primers in Table 1. Assays were performed in a volume of 50 µL, including 25 µL of AmpliTaq Gold master mix, 18 µL of ultra-purified DNAse-RNAse-free water, 1 µL of primers (20 µM of concentration) and 5 µL of DNA template. The amplification protocols were as follows: incubation step for 15 min at 95 °C, 40 cycles of one minute at 95 °C, 30 s at the annealing temperature (Table 1), an elongation step of 60–90 s at 72 °C and then a final extension step for five minutes at 72 °C. Amplifications were run in a Peltier PTC-200 model thermal cycler (MJ Research, Inc., Watertown, MA, USA) and revealed on 1.5% agarose gel. Amplicons were then purified using NucleoFast 96 PCR plates (Macherey–Nagel EURL, Hoerdt, France) as per the manufacturer’s instructions. Sequencing was conducted using the Big Dye Terminator Cycle Sequencing Kit (PerkinElmer Applied Biosystems, Foster City, CA, USA) in an ABI automated sequencer (Applied Biosystems, Waltham, MA, USA). The obtained sequences were assembled and edited using ChromasPro software (ChromasPro 1.7, Technelysium Pty Ltd., Tewantin, Australia) and compared with those available in the GenBank database by NCBI BLAST (https://blast.ncbi.nlm.nih.gov/Blast.cgi; accessed on 28 December 2021). The phylogenetic analyses, based on *16S* rRNA, *rpoB*, *FtsZ* and ITS regions of *Bartonella* spp., were inferred using neighbour-joining methods, and tree reconstructions were performed using MEGA software version 7 [13]. Bootstrap analyses were conducted using 1000 replicates.

## 3. Results

### 3.1. Rats and Ectoparasites

Twenty rodents were captured during the study period. They were all formally identified as *Cricetomys gambianus* species, the African giant pouched rat. Overall, 19 of the 20 (95%) rats carried fleas (Table S1). The number of fleas collected by brushing the rats varied from 14 to 180 per rodent. A total of 1117 fleas were recovered from the 19 rats (an average of 58.8 arthropods per rodent). Molecular identification (*18S* rRNA) confirmed the morphological identification of the fleas. All of the specimens belonged to the same species: *Xenopsylla cheopis*, the tropical rat flea, also known as the Oriental rat flea.

### 3.2. Bartonella Detection and Isolation

Regarding the 1117 tropical rat fleas collected, we selected 170 fleas (15.2%, corresponding to eight to nine specimens per rat) for screening for *Bartonella* DNA by qPCR. *Bartonella* spp. DNA was confirmed in 148 of the 170 fleas (87.1%) that were analysed. Overall, 17 *Bartonella* isolates were isolated from the blood of 17 of the 20 captured rats (prevalence of 85%). Using MALDI-TOF MS, four isolates were identified as *Bartonella massiliensis* with scores > 1.7. The other isolates were not identified to the species level, although their MS spectra undoubtedly clustered within pools of other *Bartonella* spectra.

The comparative analysis of sequences for the *16S rRNA* (1400 bp) showed that four almost similar sequences, also identified by MALDITOF-MS, exhibited a 99.9% to 100% identity with *Bartonella massiliensis* OS09 (HM636440). These strains also showed a 99–99.8%, 97.8–98.9% and 99–99.3% identity in the *FtsZ*, *ITS* and *RpoB* regions with *B*. *massiliensis*, respectively. All of the other *16S* rRNA sequences of the other isolates were close to each other and showed a 99.57% to 99.64% identity with *Bartonella elizabethae* (LR134527) and *Bartonella kosoyi* strain Tel Aviv (MN627780). They had a similarity of 95.3–96.9%, 86.7–94.5% and 92.8–96.8% with these two species at the *FtsZ*, *ITS* and *RpoB* regions, respectively. Some sequences were deleted from analysis because of their low sequencing quality (Table 2). Sequences were deposited in GenBank under accession numbers: OM458891-OM458906 for *16S*, OM459723-OM459736 for *ITS*.

**Figure 1:**
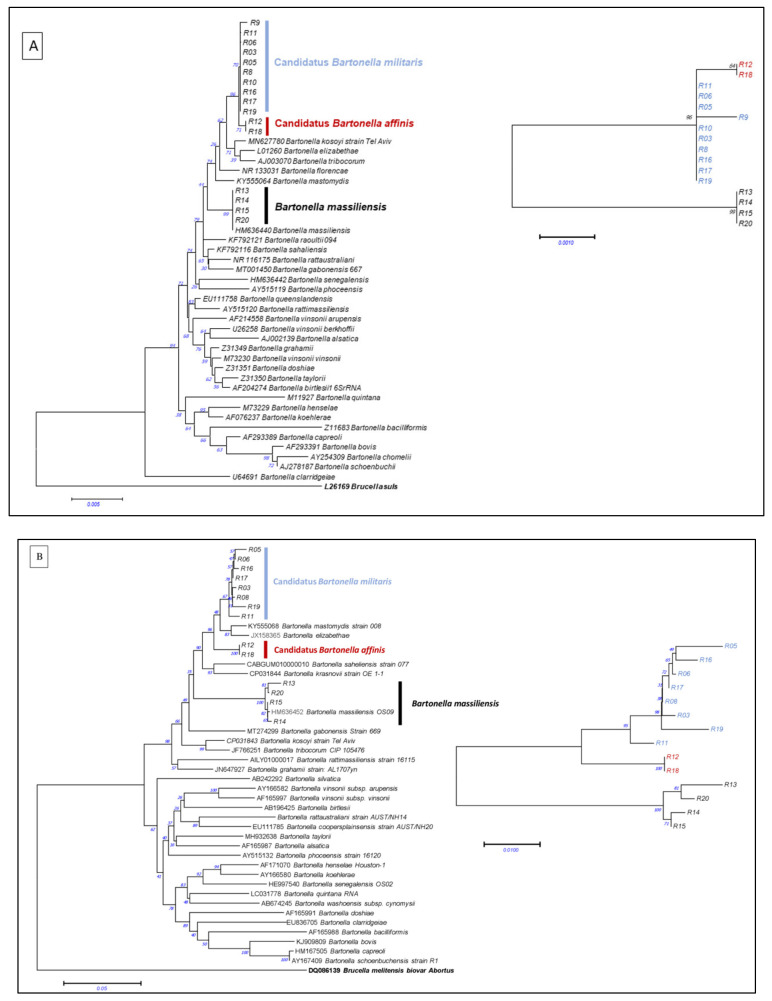

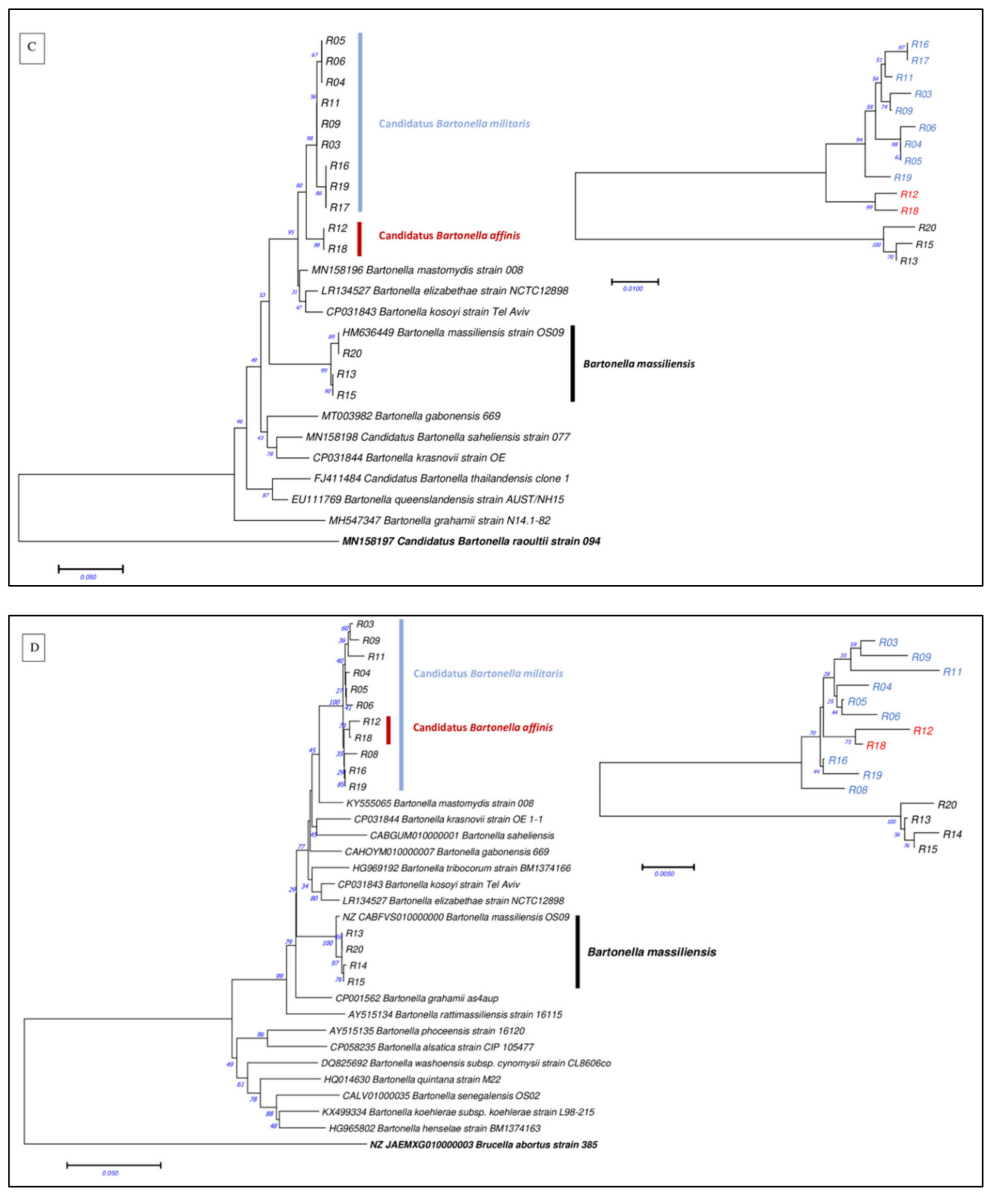
Phylogenetic analysis of *Bartonella* strains isolated in the presented study based on their genes: (**A**) *16S rRNA*, (**B**) *RpoB*, (**C**) ITS and (**D**) *FtsZ*. The topology of strains detected here is mostly similar for *16S rRNA*, *RpoB* and ITS with, in addition to strains clustered with *B*. *massiliensis*, two other separate clusters. This is not the case for the *FtsZ* gene, as we observed only two different clusters: the strains grouped with *B*. *massiliensis*, and another separate cluster performed by all of the other strains. This is well represented by the small trees, constructed only for the new sequences, for each gene. We propose two potential new species according to these phylogenies: *Candidatus* Bartonella militaris (in blue) and *Candidatus* Bartonella affinis (in red). The evolutionary history was inferred using the neighbour-joining method. The percentage of replicate trees in which the associated taxa clustered together in the bootstrap test (1000 replicates) is shown next to the branches. The tree is drawn to scale, with branch lengths in the same units as those of the evolutionary distances used to infer the phylogenetic tree. The evolutionary distances were computed using the Tamura–Nei method and are in the units of the number of base substitutions per site. The differences in the composition bias among sequences were considered in evolutionary comparisons. All positions containing gaps and missing data were eliminated. Evolutionary analyses were conducted in MEGA7 [13].

The phylogenetic analysis was based on *16S rRNA*, *RpoB* and the ITS region highlighted, in addition to four isolates clustered together with *B*. *massiliensis* and two other separate clusters. The two clusters are different from each other, as well as to the other *Bartonella* species. They are phylogenetically close to *B*. *kosoyi* and *B*. *elizabethae* or *B*. *mastomydis* (Figure 1A–C). This topology is mostly similar for *16S rRNA*, *RpoB* and ITS. This is not the case for the *FtsZ* gene, where we observed only two different clusters: the four isolates grouped with *B*. *massiliensis*, and another separate cluster performed by all of the other strains (Figure 1D).

## 4. Discussion

This study reports the isolation, molecular detection and genetic characterisation of *Bartonella* species in wild commensal rodents and their fleas from Senegal, West Africa. To our knowledge, this is the first time that *Bartonella* strains have been found in African giant rats (*Cricetomys gambianus*) and in tropical rat fleas (*Xenopsylla cheopis*) in this country.

*Bartonella* strains have been detected in rodents in several countries in Africa: Algeria, Tunisia, Egypt, Democratic Republic of Congo, Ethiopia, Tanzania, Kenya, Uganda, South Africa, Nigeria, Benin and Senegal. The infection rates reported ranged from 4% to 67% [1,8,14,15,16,17]. In Uganda, three of five *Cricetomys gambianus* trapped and examined by PCR detection and culture were *Bartonella*-positive. A genotype related to *Bartonella elizabethae* was found in one of these *Cricetomys gambianus*. Bartonellae, similar to *Bartonella massiliensis* isolated from a soft tick species *Ornithodorus sonrai* in Senegal [18], were found in two *C*. *gambianus* [19]. In West Africa, an uncultured *Bartonella sp*. was detected in 34.7% (69/199) of *X*. *cheopis* fleas collected in Cotonou, Benin [14]. *Bartonella* species were also detected in both *C*. *gambianus* blood and X. *cheopis* carried by the rats, in Nigeria [20]. In Senegal, *Bartonella* species had already been detected in ectoparasites collected from humans and animals. *Bartonella quintana* was identified in lice found on the heads of patients in Dakar [21]. Two new species within the genus *Bartonella* were isolated from rodents trapped in the Sine-Saloum region of Senegal: *B*. *saheliensis* sp. nov from the gerbil *Gerbilliscus gambianus* [22] and *B*. *mastomydis* sp. nov. from the Guinea multimammate mouse *Mastomys erythroleucus* [23]. Other new species (*B*. *senegalensis* sp. nov and *B*. *massiliensis* sp. nov) were also found in the soft tick *Ornithodoros sonrai*, the vector of relapsing fever [18].

In this study, the prevalence of *Bartonella* is very high, even regarding previous reports on rats and their ectoparasites. In 2017, a study reported a collection of 407 fleas from small mammals trapped around homes in five villages, in north-western Uganda. Among these fleas, 202 (49.6%) were identified as *X*. *cheopis*, mainly from *Rattus rattus* (*n* = 148), the black rat. The *Bartonella* prevalence in oriental rat fleas collected from all of the rodents was relatively low (9%; 18/202). The phylogenetic relationships of *Bartonella* genetic variants, identified from sequences obtained by analysing fleas, showed the infection by variants partly belonging to the *B*. *elizabethae* species complex [17]. In Tanzania, another study reported the presence of *Bartonella* DNA in 27.5% (53/193) of *X*. *cheopis* fleas collected from *Rattus rattus* specimens. The genotypes were closely related to different *Bartonella* species, including *B*. *elizabethae* [1].

In our study, the high number (*n* = 1117) of *Xenopsylla cheopis* collected from only 20 *C*. *gambianus* suggests a large infestation of the African giant rats by fleas in the location studied and, potentially, in the surroundings. The flea *X*. *cheopis* is a well-known parasite of rodents, primarily of the genus *Rattus*, but can live on many warm-blooded mammals. Moreover, this flea species is a vector for bubonic plague, caused by *Yersinia pestis*, and can transmit *Rickettisa felis*, the aetiological agent of murine typhus [24]. The high *Bartonella* prevalence in this study can, first, confirm the role of *C*. *gambianus* and *X*. *cheopis* as reservoirs and vectors, respectively. Second, this high prevalence can be due to the infestation rate of rats (around 55.8 fleas per rat): this facilitates the transmission to and between rats and the reinfection of fleas in return. It seems that the high infection rate in rats conduces to a high detection rate in fleas feeding on these rats. Other arguments are needed to demonstrate that fleas can maintain these *Bartonella* spp. (such as the persistence of flea infection).

The isolated strains from rats are, in general, close to the *Bartonella* species previously described or detected in rodents such as *B*. *elizabethae*, *B*. *kosoyi* or *B*. *mastomydis*; or from rodent-ectoparasites, as in the case of *B*. *massiliensis*. Here, we isolated *B*. *massiliensis* in rats. The adaptation of *B*. *massiliensis* to both rodents and their ectoparasites has a great impact in terms of its epidemiologic dynamism. It may promote the spread of this agent and its colonisation of other hosts. In addition, two potential new species, phylogenetically related to zoonotic *B*. *elizabethae*, as well as to other rodent-associated *Bartonella*, were identified based on the *Bartonella* gene-sequence-based criteria for species definition [25]. Here, we propose preliminary names for these genogroups, namely (i) *Candidatus* Bartonella militaris and (ii) *Candidatus* Bartonella affinis (Figure 1). The genetic difference was clear between these two species basing on *16S*, *RpoB* and ITS, whereas *FtsZ* did not show this difference, which required the use of more than one target to identify bacteria belonged to the bartonella genus. The high *Bartonella* infection rate in rodents and their ectoparasites, observed here and in the previous studies, could explain this emergence of new species. However, the role and/or pathogeny of this species in humans or animals remain to be explored. Since our team began studying *Bartonella* in rodents over the past decade, at least seven species have been isolated and described, not counting these two species: *B*. *florenciae*, *B*. *mastomydis*, *B*. *massiliensis*, *B*. *senegalensis*, *B*. *saheliensis*, *B*. *gabonensis* and *B*. *raoultii* [18,22,23,26,27,28,29]. The risks that they pose to human and animal health remain unknown.

In addition, due to its size and weight, the African giant rat is considered as a natural resource of animal protein by the local population [7]. The prevalence of *Bartonella* sp. found in this study may alert local sanitary authorities to the public health risk for hunters or breeders, but also for inhabitants, given that rat holes have been observed in residential areas, especially in semi-urban locations.

The potential new species described in this study are related genetically to *B*. *elizabethae*, a species that is currently recognised as a zoonotic agent responsible for endocarditis [30], neuroretinitis [30,31] and bacillary angiomatosis [32] in humans. Veterinarians also consider this bacterium as an infectious disease agent in canine medicine [33]. In Senegal, only a few studies have reported clinical cases of bartonellosis with identification of the infectious agent. One case of endocarditis caused by *B*. *quintana* was described in a Senegalese patient [34] in 2002. More recently, an investigation showed the circulation of *B*. *quintana* in febrile patients visiting a health facility in rural Senegal [35].

This study helps to confirm that *X*. *cheopis* commonly carry the bacteria of the genus *Bartonella* and potentially play an important role in the transmission of potential zoonotic infections among Gambian pouched rats and from rats to humans. Due to the nature of the *Bartonella* species detected, this study shows the importance, from a public health point of view, of leading further investigations into the potential zoonotic *Bartonella* species in Senegal and, more generally, in Africa. Future research may include more epidemiological studies on pests and farm animals due to the close and increasing interaction between humans, animals and the environment. The new potential species merit a complete description, including their genomic features, due to their microbiological, epidemiological and pathological characteristics being unknown to date.

## Figures and Tables

**Table 1:** Primers and probes used in the present study for the molecular investigations and sequencing.

PCRs	Target Genes	Primer Names	Sequences	References
Screening by qPCR	*ITS3, Bartonella* spp. *(Intergenic 16S-23S)*	Barto_ITS3_F	GATGCCGGGGAAGGTTTTC	[10]
		Barto_ITS3_R	GCCTGGGAGGACTTGAACCT	
		Barto_ITS3_P	6FAM-GCGCGCGCTTGATAAGCGTG	
	*ITS2, Bartonella* spp. *2nd intention*	Barto_ITS2_F	GGGGCCGTAGCTCAGCTG	
		Barto_ITS2_R	TGAATATATCTTCTCTTCACAATTTC	
		Barto_ITS2_P	6FAM-CGATCCCGTCCGGCTCCACCA	
Standard PCRs and sequencing	*16S, Bacteria*	Fd1	AGAGTTTGATCCTGGCTCAG	[11]
		Rp2	ACGGCTACCTTGTTACGACTT	
	*ITS, Bartonella* spp.	Urbarto1	CTTCGTTTCTCTTTCTTCA	[12]
		Urbarto2	CTTCTCTTCACAATTTCAAT	
	*ftsZ, Bartonella* spp.	FTSZDIR	CCGTGAATAATATGATTAATGC	
		FTSZREV	TTGAAATGGCTTTGTCACAAC	
	*rpoB, Bartonella* spp.	1400F	CGCATTGGCTTACTTCGTATG	
		2300R	GTAGACTGATTAGAACGCTG	
		1596R	GGACAAATACGACCATAATGCG	
		2028F	GGAAAATGATGATGCGAATCGTGC	
		1873R	TCYTCCATMGCWGAMAGATAAA	

**Table 2:** Identities and sizes of the generated sequences for *Bartonella* isolates collected in the present study.

Isolates	Blast Results: Identity (%) and Size (bp) for the Sequenced Genes								
	Best Results	*16S*	Size	*RpoB*	Size	*ITS*	Size	*FtsZ*	Size
R03	*B. kosoyi strain Tel Aviv (MN627780), B. elizabethae strain NCTC12898 (LR134527)*	99.6	1400	93.2–96.3	1001	87.9–94.1	727	96.2–96.3	894
R04	*B. kosoyi strain Tel Aviv (MN627780), B. elizabethae strain NCTC12898 (LR134527)*	-	-	-	-	87.7–89.9	739	95.6–96	889
R05	*B. kosoyi strain Tel Aviv (MN627780), B. elizabethae strain NCTC12898 (LR134527)*	99.6	1408	93.4–96.2	866	88–89.9	730	96–96.2	906
R06	*B. kosoyi strain Tel Aviv (MN627780), B. elizabethae strain NCTC12898 (LR134527)*	99.6	1416	93.4–96.8	868	87.2–89	781	95.3–95.6	900
R08	*B. kosoyi strain Tel Aviv (MN627780), B. elizabethae strain NCTC12898 (LR134527)*	99.6	1401	93.7–96.5	898	-	-	95.9–96.1	879
R09	*B. kosoyi strain Tel Aviv (MN627780), B. elizabethae strain NCTC12898 (LR134527)*	99.6	1400	-	-	88.8–90	708	95–95.4	895
R10	*B. kosoyi strain Tel Aviv (MN627780), B. elizabethae strain NCTC12898 (LR134527)*	99.6	1400	-	-	-	-	-	-
R11	*B. kosoyi strain Tel Aviv (MN627780), B. elizabethae strain NCTC12898 (LR134527)*	99.6	1351	92.8–95.7	906	86.7–94.5	727	95.4–95.8	877
R12	*B. kosoyi strain Tel Aviv (MN627780), B. elizabethae strain NCTC12898 (LR134527)*	99.6	1400	-	-	88.8–91.7	715	95.3–95.5	925
R13	*B. massiliensis strain OS09 (HM636440)*	100	1401	99	877	98.3	786	99.5	868
R14	*B. massiliensis strain OS09 (HM636440)*	100	1401	99.2	874	-	-	99.1	895
R15	*B. massiliensis strain OS09 (HM636440)*	99.9	1401	99.3	1018	97.8	770	99.8	875
R16	*B. kosoyi strain Tel Aviv (MN627780), B. elizabethae strain NCTC12898 (LR134527)*	99.6	1400	93.8–96.7	873	87.9–90.2	737	96.4–96.7	913
R17	*B. kosoyi strain Tel Aviv (MN627780), B. elizabethae strain NCTC12898 (LR134527)*	99.6	1400	93.8–96.7	868	87.5–89.6	731	-	-
R18	*B. kosoyi strain Tel Aviv (MN627780), B. elizabethae strain NCTC12898 (LR134527)*	99.6	1410	94–95.9	889	89.6–91.5	720	95.9–96	909
R19	*B. kosoyi strain Tel Aviv (MN627780), B. elizabethae strain NCTC12898 (LR134527)*	99.6	1400	93.5–96.2	865	88.3–90.4	771	96.6–96.9	915
R20	*B. massiliensis strain OS09 (HM636440)*	100	1402	99	868	98.9	812	99.1	902

## Supplementary Materials

The following supporting information can be downloaded at: https://www.mdpi.com/article/10.3390/microorganisms10030489/s1, Table S1. Details on rat and flea samples, qPCR results and culture.
